# Evidence of a Lytic Pathway in an Invertebrate Complement System: Identification of a Terminal Complement Complex Gene in a Colonial Tunicate and Its Evolutionary Implications

**DOI:** 10.3390/ijms252211995

**Published:** 2024-11-08

**Authors:** Loriano Ballarin, Anna Peronato, Davide Malagoli, Paolo Macor, Sandro Sacchi, Gabriele Sales, Nicola Franchi

**Affiliations:** 1 Department of Biology, University of Padova, 35131 Padova, Italy; loriano.ballarin@unipd.it (L.B.);; 2 Department of Life Sciences, University of Modena and Reggio Emilia, 41125 Modena, Italysandro.sacchi@unimore.it (S.S.); 3 NBFC, National Biodiversity Future Center, 90133 Palermo, Italy; 4 Department of Life Sciences, University of Trieste, 34127 Trieste, Italy

**Keywords:** complement system, lytic pathway, terminal complement complex, C9, invertebrate, complement system evolution

## Abstract

The complement system is a pivotal component of innate immunity, extensively studied in vertebrates but also present in invertebrates. This study explores the existence of a terminal complement pathway in the tunicate *Botryllus schlosseri*, aiming to understand the evolutionary integration of innate and adaptive immunity. Through transcriptome analysis, we identified a novel transcript, BsITCCP, encoding a protein with both MACPF and LDLa domains—a structure resembling that of vertebrate C9 but with a simpler organization. Phylogenetic reconstruction positions BsITCCP between invertebrate perforins and vertebrate terminal complement proteins, suggesting an evolutionary link. Localization studies confirmed that *bsitccp* is transcribed in cytotoxic morula cells (MCs), which are also responsible for producing other complement components like BsC3, BsMBL, BsMASP, and BsBf. Functional assays demonstrated that *bsitccp* transcription is upregulated in response to nonself challenges and is dependent on BsC3 activity; inhibition of BsC3 led to a significant reduction in BsITCCP expression. Electron microscopy revealed that MCs form contact with perforated yeast cells, indicating a possible mechanism of cell lysis similar to the immunological synapse observed in vertebrates. These findings suggest that a C3-governed lytic complement pathway exists in *B. schlosseri*, challenging the assumption that a C5 ortholog is necessary for such a pathway. This work enhances our understanding of the evolution of the complement system and suggests that invertebrates possess a terminal complement complex capable of mediating cell lysis, regulated by C3. Future studies will focus on confirming the pore-forming ability of BsITCCP and its role in the immunological synapse.

## 1. Introduction

The complement system represents the main soluble component of innate humoral immunity [1,2]. This statement, based on evidence gathered over the years from studying vertebrates, can now be extended to invertebrates, where the basic components of the complement system have been described in almost all phyla [3,4].

In mammals, we know more than 50 proteins involved in the classical, lectin, and alternative activation pathways, as well as in the terminal lytic pathway, along with many regulatory proteins [5]. In invertebrates, conversely, we know only some of these components as parts of the alternative and lectin pathways [6].

Today, it is widely accepted that C3, the central complement protein, appeared more than 800 million years ago in Cnidarians and that the alternative pathway, involving the proteolytic cleavage of C3 generating C3a and C3b, is the oldest complement activation pathway [7]. The lectin pathway appeared more recently, whereas the classical pathway, which depends on the presence of antibodies, emerged only with the Gnathostomes [8].

An important consideration is that, in the course of vertebrate evolution, a first genomic duplication occurred, followed by an additional one when Gnathostomes appeared [9]. Therefore, it is now clear that C5 originated from gene duplication (along with C4) from the common ancestor C3 at the basis of vertebrate evolution. As for the lytic pathway (also called the terminal complement complex, TCC), today we now know that it relies on six proteins derived from a single common ancestor through gene duplication: C6, C7, C8α, C8β, C8γ, and C9. In particular, C6, interacting with C5b, initiates the assembly of the membrane attack complex (MAC), allowing the binding of C7, followed by C8s, and finally C9. C9 is the protein that forms a pore in the lipid bilayer and leads to the lysis of the target cell [2,5].

Vertebrate C6-9 proteins share the MACPF domain, also shared by perforins, older proteins widely distributed throughout the animal kingdom [10]. What varies among the various TCC components is the presence of accessory domains such as TSP1, LDLa, EGF, CCP, and FIMAC [5]. Among the above proteins, C6 possesses all the aforementioned domains, some in multiple copies, while the composition of domains is progressively less complex in C7, C8, and C9, the latter being the simplest component with an architecture closely resembling that of perforins [11,12].

Unfortunately, to date, there are no studies in invertebrates investigating the real presence of a lytic pathway controlled by C3, as it has been accepted that the ancestral gene of the pathway should have been a C6-like gene [2,5]. This hypothesis was initially proposed in a 1993 paper by Hobart and colleagues [13], who postulated that the ancestral gene should have presented a high complexity in domain organization, which could then be lost following duplications, although they were aware that “There is a widely held assumption that evolution proceeds from the simple to the complex”.

Following the above assumption, researchers continued to search for a common ancestor of the lytic pathway resembling C6 without ever finding it. Instead, in early investigations based on the genome projects of various invertebrates, genes were found coding for simpler proteins that presented only the MACPF domain and few other domains. As they lacked an architecture comparable to C6 and since invertebrates do not have a gene for C5, it was quickly concluded that invertebrates do not possess a terminal complement complex (TCC) [2].

The compound ascidian *Botryllus schlosseri* is a reliable model organism for the study of immunobiology [14,15]. Its immunocytes represent the great majority of the circulating hemocytes. In this species, we already demonstrated the presence of the lectin and the alternative complement pathways, with the identification of homologs of mammalian C3, Bf, MBL, and MASP1, referred to as BsC3, BsBf, BsMBL, and BsMASP, respectively [3,16]. All the complement components identified so far are expressed by morula cells (MCs), the most abundant circulating hemocytes (40–60% of the total hemocytes) [3,17]. In this type of cell, we have also demonstrated the presence of a C3a/C5a receptor. Therefore, it seems plausible that the activation of BsC3a induces *bsc3* overexpression in *B. schlosseri* MCs by acting on its specific receptor [15], suggesting the existence of a positive autocrine feedback loop where BsC3 is induced by the MCs themselves.

In this study, by mining the available transcriptome, we identified, in *B. schlosseri*, a new transcript containing both a MACPF and an LDLa domain, which we named BsITCCP because we consider it to be a member of the newly identified family of Invertebrate Terminal Complement Complex Proteins (ITCCPs). We investigated its localization through in situ hybridization and immunocytochemistry. Additionally, we studied its expression in the presence of zymosan, as an immune challenge, with or without C3 inhibition, demonstrating that the presence of the *bsittcp* transcript and protein is impaired when BsC3 is either blocked by a specific antibody or inhibited by compstatin.

## 2. Results

### 2.1. Subsection

#### 2.1.1. BsITCCP Gene, Transcript, Protein and 3D Structure

**Figure 1:**
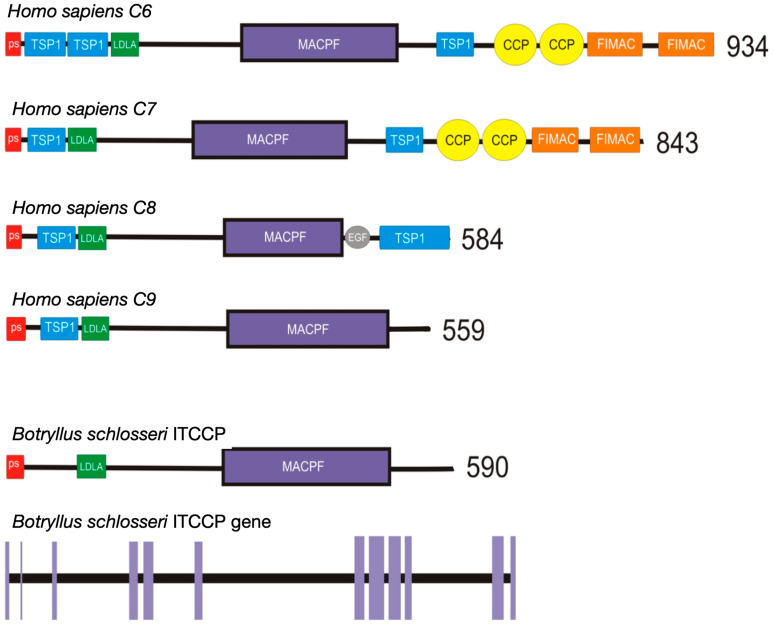
Domain organization in human complement components 6 (C6), 7 (C7), 8 (C8), and 9 (C9) compared with the domain organization of the ITTC protein of B. schlosseri and the gene organization of BsITCCP where exons are represented by purple lines.

By BLAST analysis of our *B. schlosseri* EST collection and 3′ RACE, we identified a unique transcript for a protein with a MACPF domain. The protein was named BsITCCP, identifying it as a member of the invertebrate terminal complement complex. The transcript sequence was deposited in Genbank under the accession number PQ126347.1 and was confirmed by alignment with the *B. schlosseri* genome. The gene organization, the transcript sequence, and the comparison in domain organization of the putative protein with the components of the terminal complement complex in humans are reported in Figure 1 and Table S1. The transcript codes for a protein of 590 amino acids, with a molecular weight of 65.17 kDa and a signal peptide of 22 residues. The MACPF domain is 219 amino acids long.

**Figure 2:**
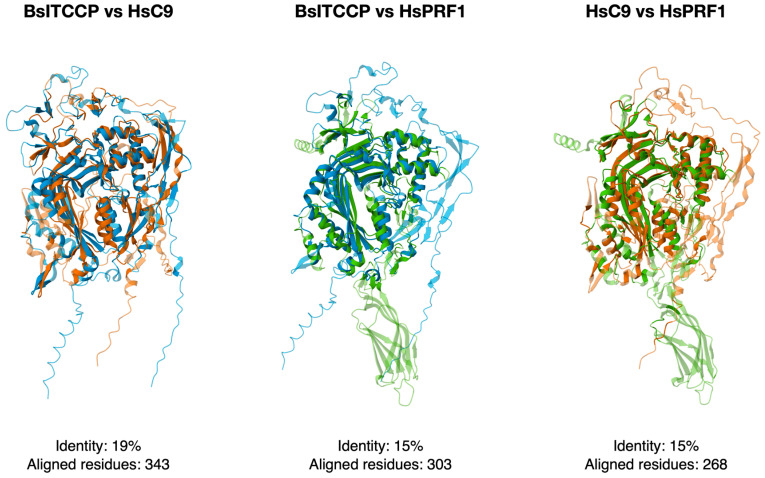
Predicted 3D structure of BsITCCP (blue) as compared with human C9 (orange) and human PRF1 (green).

The predicted structure of BsITCCP supports its classification as a protein comparable to the vertebrate C9 protein family. A comparison of BsITCCP with human C9, as shown in Figure 2, reveals a significant degree of similarity in the arrangement of their respective 3D structures, with many BsITCCP domains aligning closely with those of the human protein. The structural comparison between the two human paralogous genes, HsC9 and HsPRF1 (the typical perforin found in NK cells of mammals), shows lower identity and amino acid alignment scores compared to the HsC9 and BsITCCP comparison. In contrast, the comparison between BsITCCP and HsPRF1 shows scores comparable to those of the two human proteins (Figure 2).

#### 2.1.2. In the Presence of Zymosan, BsITCCP Increases Its Transcription at a Slower Rate Than BsC3

**Figure 3:**
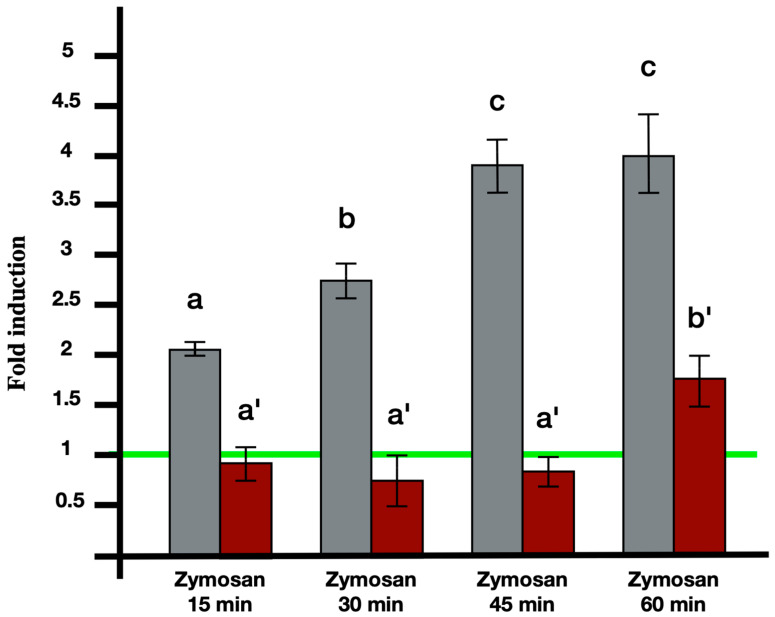
RT-PCR analysis showing the increase in the transcription of bsc3 (grey columns) and bsitccp (red columns) from hemocytes treated with 0.1 mg/mL Zymosan for 15, 30, 45, and 60 min. Untreated control samples are set to 1 (green line). Histogram bars with different letters represent statistically significant differences between them, with *p* < 0.05.

**Figure 4:**
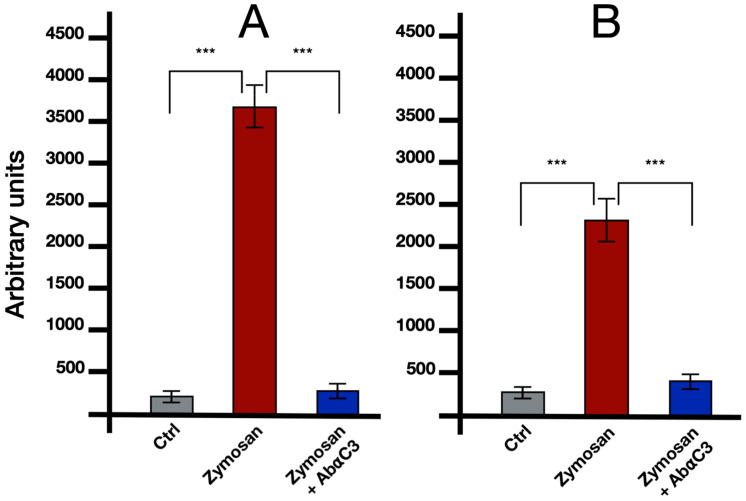
RP-PCR analysis for BsC3 (**A**) and BsITCCP (**B**) transcripts from microinjected colonies treated with 0.1 mg/mL Zymosan and 0.1 mg/mL Zymosan plus antibodies against human C3 and sacrificed after 6 h. Gray columns: control colonies, injected with FSW and anti-C3 antibody; red columns: colonies treated with zymosan 0.1 mg/mL; blue columns: colonies microinjected with zymosan 0.1 mg/mL and anti-C3 antibody. Asterisks mark significant differences with respect to the controls. ***: *p* < 0.001.

In experiments involving induction with 0.1 mg/mL zymosan microinjected into the colonies and left to act for 6 h, both BsITCCP and BsC3 were significantly overexpressed. The semi-quantitative RT-PCR analysis on hemocytes indicates that BsITCCP remains at control levels up to 45 min and then increases significantly (*p* < 0.05) at 60 min, showing a twofold expression as compared to the controls (Figure 3). In contrast, BsC3 is modulated within a short time frame, being significantly (*p* < 0.05) upregulated as early as 15 min, and its transcription continues to increase at 30 and 45 min. BsC3 remains highly expressed at 60 min, similar to the level observed at 45 min, showing a fourfold increase compared to the controls. Conversely, the levels of both transcripts remain at control levels in colonies where, in addition to zymosan, the anti-C3 antibody was also injected (Figure 4).

#### 2.1.3. The Anti-hC9 Antibody Recognized a Single Band of Approximately 59 kDa in Homogenates of *Botryllus* Colonies

**Figure 5:**
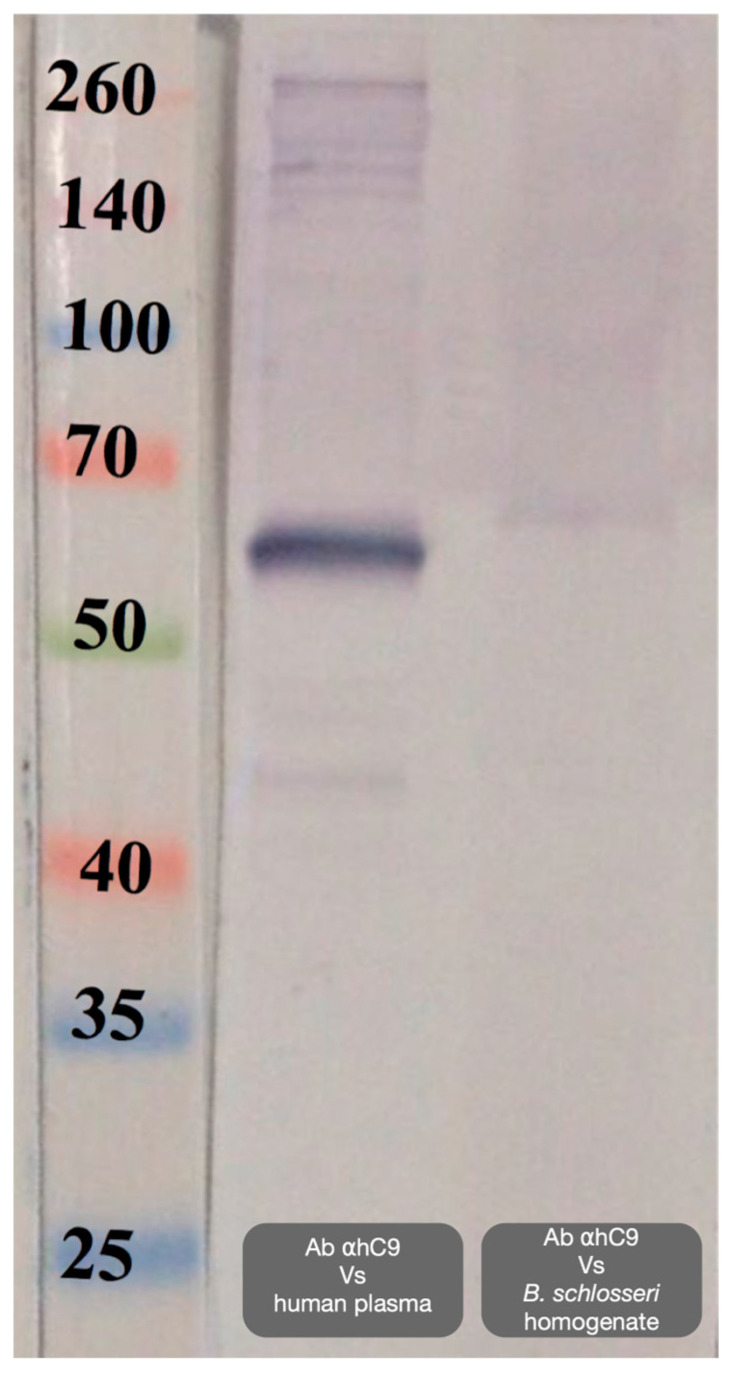
Western blot with antibodies anti-human C9 on *B. schlosseri* protein lysates and human plasma.

The anti-hC9 antibody recognized a distinct band slightly above 50 kDa in lysates from *B. schlosseri* hemocytes and human plasma. The predicted MW corresponds to the expected size of BsITCCP (Figure 5). 

#### 2.1.4. BsITCCP Is Localized in Cytotoxic MCs and the Number of Positive Cells Increases After Treatment with Zymosan

**Figure 6:**
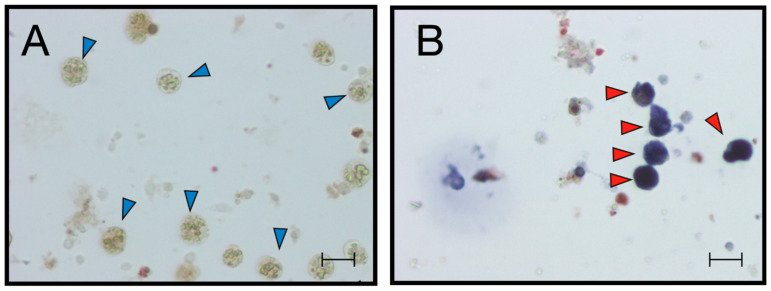
ISH on hemocytes (**A**) sense probe for BsITCCP; (**B**) antisense probe for BsITCCP. Scale bar: 10 μm. Red arrow: labeled MCs, blue arrow: unlabeled MCs.

**Figure 7:**
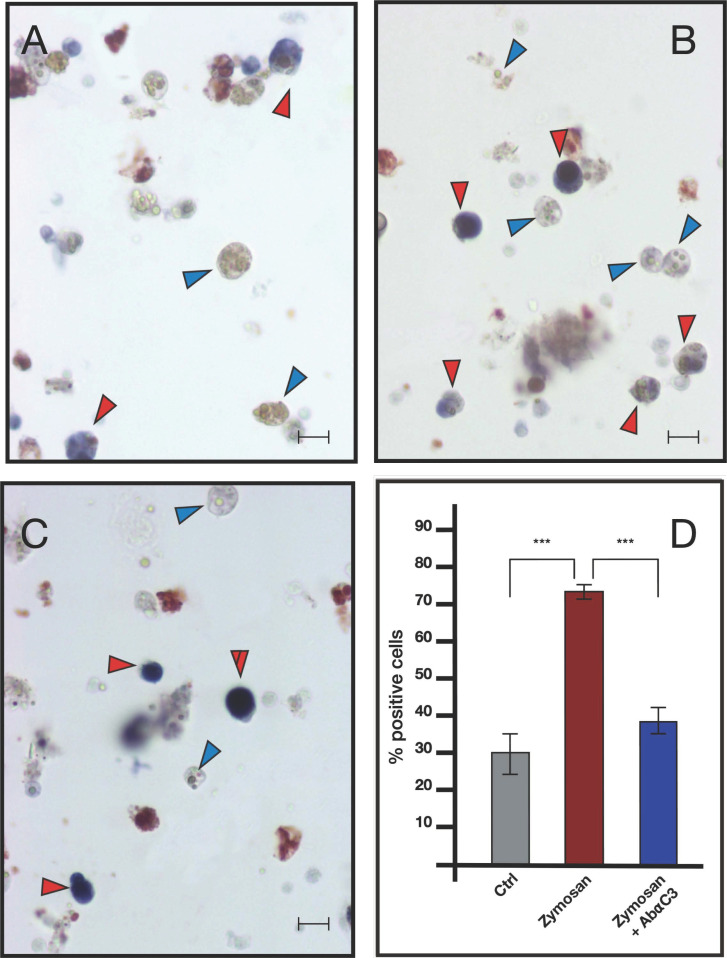
ISH with BsC3 antisense probe on hemocytes treated with 0.1 mg/mL Zymosan and 0.1 mg/mL Zymosan plus antibodies against human C3. (**A**) Control cells; (**B**) cells treated for 6 h with zymosan 0.1 mg/mL; (**C**) cells treated for 6 h with zymosan 0.1 mg/mL and anti-hC3 antibody; (**D**) percentage of marked hemocytes in the various treatments. Red arrow: labeled MCs, blue arrow: unlabeled MCs. Scale bar: 10 μm. *** *p* < 0.001.

**Figure 8:**
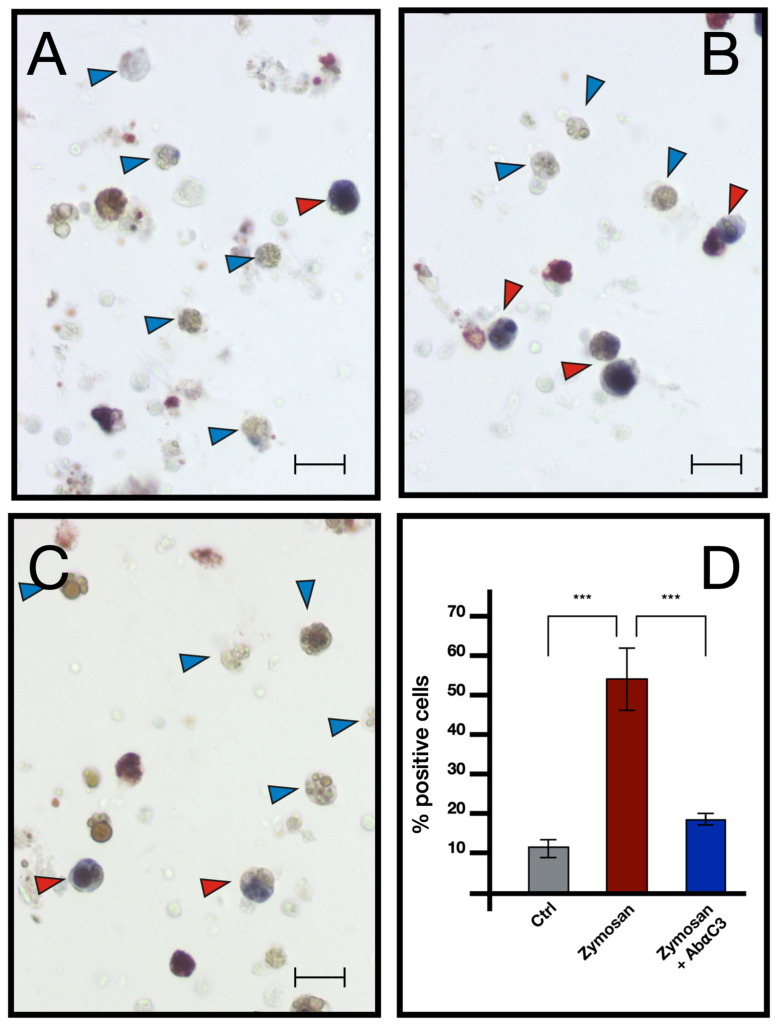
ISH with BsITCCP antisense probe on hemocytes treated with 0.1 mg/mL Zymosan and 0.1 mg/mL Zymosan plus antibodies against human C3. (**A**) Control cells; (**B**) cells treated for 6 h with zymosan 0.1 mg/mL; (**C**) cells treated for 6 h with zymosan 0.1 mg/mL and anti-hC3 antibody; (**D**) percentage of marked hemocytes in the various treatments. Red arrow: labeled MCs, blue arrow: unlabeled MCs. Scale bar: 10 μm. *** *p* < 0.001.

ISH analysis indicated that MCs are the only cells transcribing bsitccp (Figure 6). In our previous paper [17] we demonstrated that this is also true for BsC3. The fraction of cells labeled by the antisense probe for BsC3 and BsITCCP significantly (*p* < 0.001) increased with respect to the controls after 6 h of zymosan treatment. The fraction of positive cells remained similar to the control values when cells were incubated with both zymosan and the anti-hC3 antibody (Figure 7 and Figure 8).

**Figure 9:**
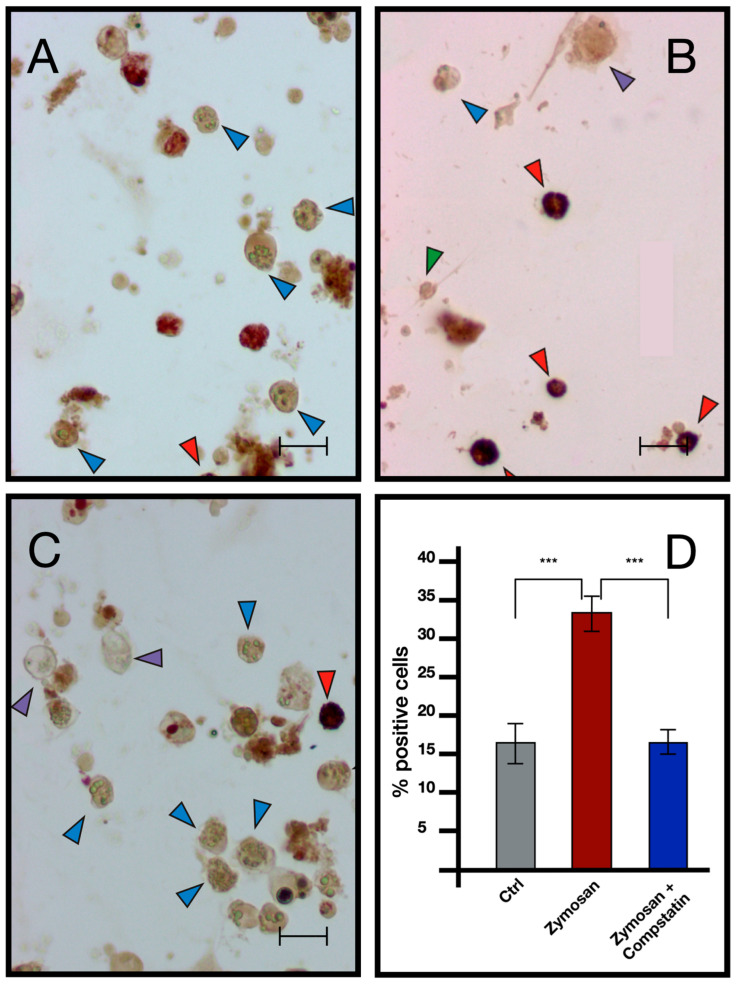
Immunocytochemistry with anti-C3 antibody on hemocytes untreated and treated with 0.1 mg/mL Zymosan and 0.1 mg/mL Zymosan plus Compstatin. (**A**) Control in FSW; (**B**) treated for 6 h with Zymosan 0.1 mg/mL; (**C**) treated for 6 h with Zymosan 0.1 mg/mL + Compstatin 100 μM; (**D**) percentage of marked hemocytes under the previously described conditions. Red arrow: marked morula cells, blue arrow: unmarked morula cells, purple arrow: phagocytes, green arrow: hyaline amebocytes. Scale bar: 10 μm. *** *p* < 0.001.

**Figure 10:**
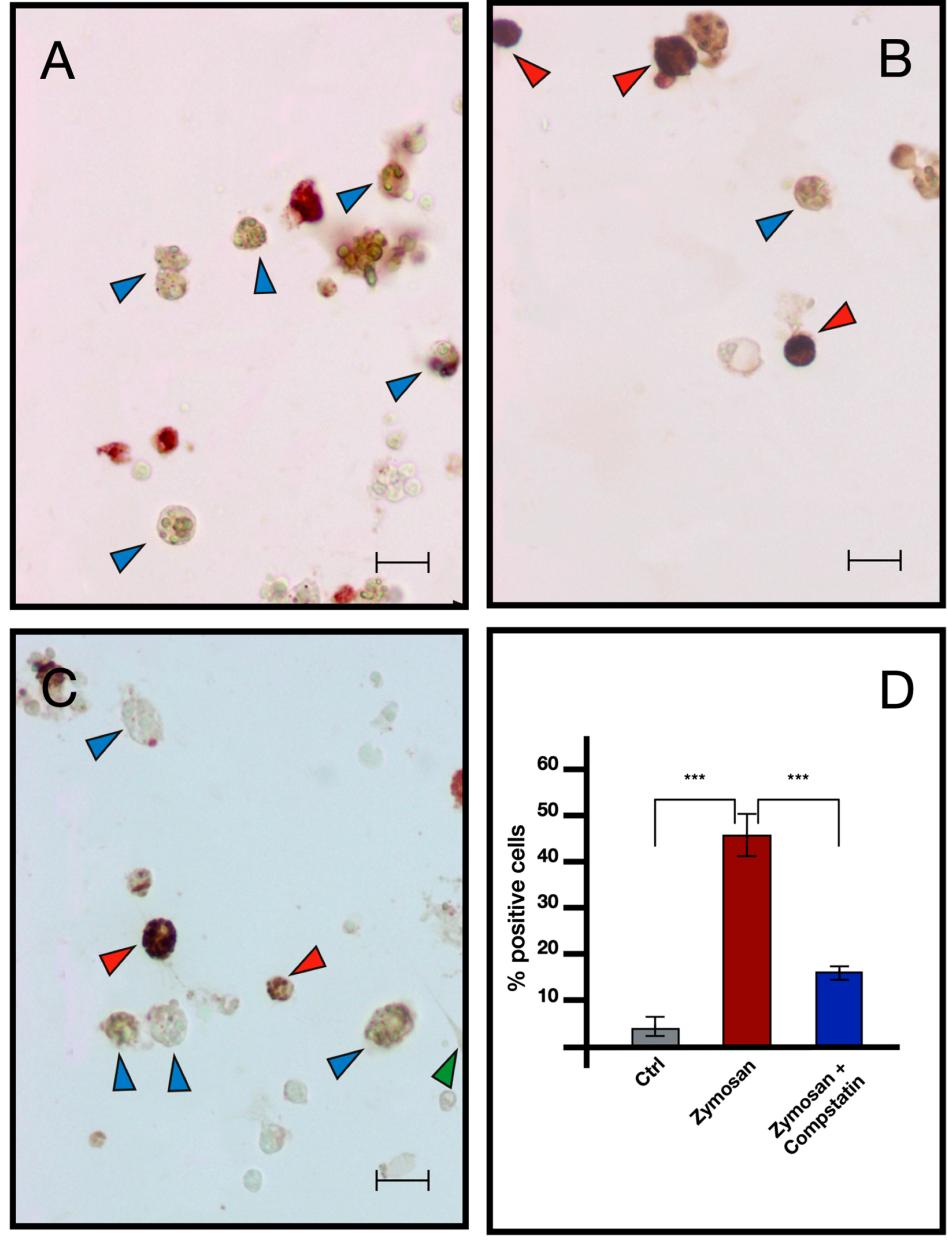
Immunocytochemistry with anti-hC9 antibody on *B. schlosseri* hemocytes treated with 0.1 mg/mL Zymosan and 0.1 mg/mL Zymosan plus Compstatin. (**A**) Control cells incubate in FSW; (**B**) cells treated for 6 h with zymosan 0.1 mg/mL; (**C**) cells treated for 6 h with zymosan 0.1 mg/mL + compstatin 100 μM; (**D**) percentage of marked hemocytes. Red arrow: marked morula cells, blue arrow: unmarked morula cells, green arrow: hyaline amebocytes. Scale bar: 10 μm. ***: *p* < 0.001.

Comparable results emerged from immunocytochemical analysis, where the number of cells showing immunopositivity to the anti hC3 and the hC9 antibodies increases significantly in samples treated with zymosan for 6 h but remains at control levels in samples treated with both zymosan and the drug compstatin, a C3 inhibitor, effective on both human and *B. schlosseri* C3, as previously demonstrated in our earlier publication [16] (Figure 9 and Figure 10).

#### 2.1.5. Phylogenetic Analyses

**Figure 11:**
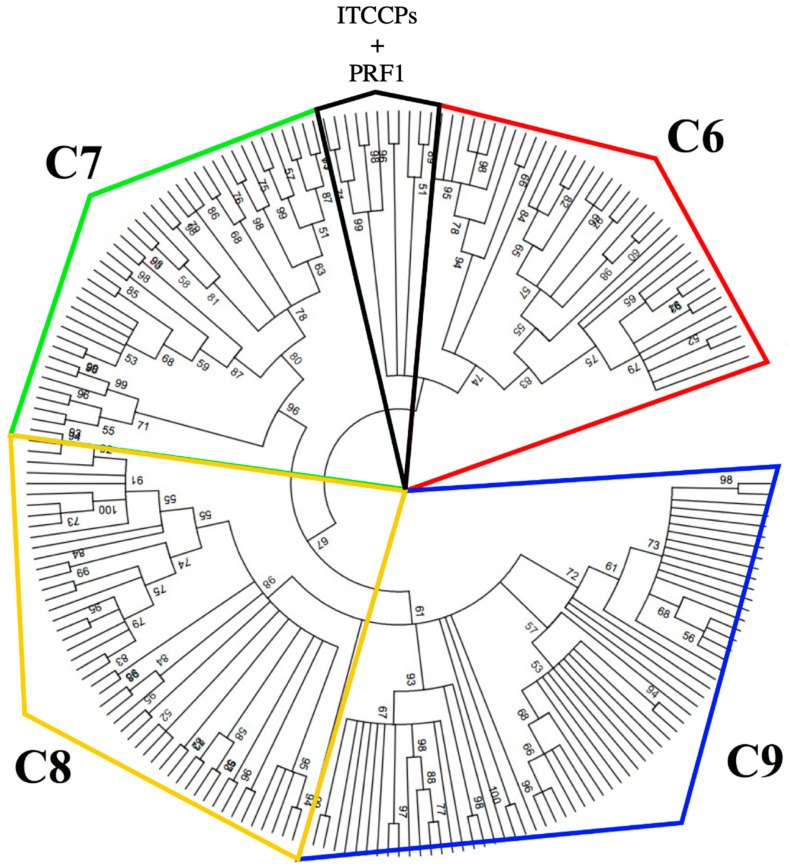
Phylogenetic tree of the MACPF domain for C6, C7, C8, C9, ITCCP, and PRF1 proteins, calculated using the maximum likelihood method with 2000 bootstrap replicates. Schematic representation.

The phylogenetic tree of the MACPF domain obtained with the maximum likelihood (ML) showed the presence of four main clusters, one represented by vertebrate C6 proteins with the ITCCP sequences from Tunicates and perforins (PRF1) from vertebrates, and others containing C7, C8, and C9 from vertebrates, respectively. The clusters of C6, C7, C8, and C9 are well resolved with bootstrap values above 50, while the invertebrate group remains unresolved (Figure 11 and Figure S1).

#### 2.1.6. MCs Can Closely Adhere to Yeast Cells

**Figure 12:**
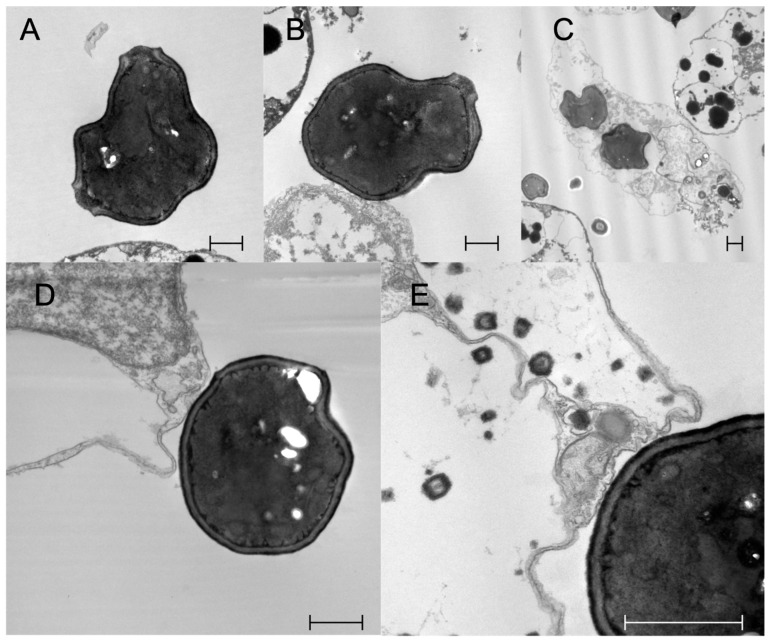
Transmission electron micrographs of hemocyte cells from *B. schlosseri* incubated with Saccharomyces cerevisiae (Yeast). (**A**,**B**) Yeast cells with evident signs of lysis near an MC; (**C**) yeast cell engulfed by a phagocyte; (**D**,**E**) close contact between an MC and a yeast cell. Scale bars: 1 μm.

TEM analysis of *B. schlosseri* hemocytes incubated with yeast cells revealed that the plasma membrane of some MCs contacts the yeast cell wall, forming a junction that morphologically resembles the immunological synapse formed by mammalian NK cells with target cells to facilitate the action of perforins (Figure 12D,E). Lysed yeast cells were found outside the MCs (Figure 12A–C).

## 3. Discussion

In recent years, our research group has focused on describing the main components of the complement system in *B. schlosseri* [3,14,15,16,18] to provide new data to better elucidate the evolution of the complement system and understand how adaptive immunity, which appeared with the emergence of Gnathostomes, integrated with the older innate immunity of which the complement system is a part.

Tunicates, as the sister group of vertebrates, represent excellent model organisms for comparative immunology studies, particularly for those addressed to clarify the emergence of new molecular pathways in vertebrates [19]. Up to now, it remains unknown whether, in addition to the lectin and alternative pathways of C3 activation, invertebrates also possess a downstream lytic pathway that leads to cell lysis under the control of C3.

By searching for sequences containing the MACPF domain in the available transcriptomes, we found that, in *B. schlosseri*, there is only one sequence that contains this domain, and it has a very simple organization with an LDLa domain at the N-terminal and a signal peptide. Such an organization strongly resembles that of the C6–C9 protein family of vertebrates, particularly C9, which is the protein with the simplest domain organization, similar to that of perforins [5]. The 3D structural alignment of BsITCCP with HsC9 and HsPRF1 further highlights that the protein shape of *B. schlosseri* more closely resembles that of HsC9, while still displaying a significant degree of similarity with HsPRF1. This same level of structural similarity is also observed between the two human paralogous genes, C9 and PRF1. BsITCCP, unlike vertebrate C9, lacks the TCP1 domain. The thrombospondin type 1 repeat (TSP1) domain is present in thrombospondins, where it functions as a regulator of cell interactions in vertebrates (InterPRO description: IPR000884). Its presence in vertebrate C9 may be related to the need for this protein to interact with other proteins of the terminal complement complex (TCC). The absence of this domain in the *B. schlosseri* sequence, therefore, could support the idea that such interactions are not required and that BsITCCP may polymerize in the membrane without needing to interact with other proteins.

Trying to reconstruct a phylogenetic relationship among the MACPF-containing sequences, we decided to exclude the accessory domains and focus only on the MACPF domain, as it is present in all the members of the vertebrate terminal complement complex and the vertebrate and invertebrate perforins [20,21,22,23]. Our phylogenetic reconstruction leads us to conclude that MACPF-containing sequences in invertebrate chordates group with those of vertebrate perforins and, secondarily, with vertebrate C6 proteins. Based on this evidence, we conclude that there is a protein in *B. schlosseri*, with the MACPF domain that is phylogenetically positioned between the vertebrate perforins and the vertebrate terminal complement complex proteins. This protein, which we named BsITCCP (Invertebrate Terminal Complement Complex Protein), even though clustering with vertebrate C6, maintains the domain organization typical of the complement proteins capable of oligomerizing to form pores in membranes, a feature lacking in C6, C7, and C8 but conserved by C9 [24]. Indeed, the anti-hC9 antibody recognized a band corresponding to the expected MW of BsITCCP.

**Figure 13:**
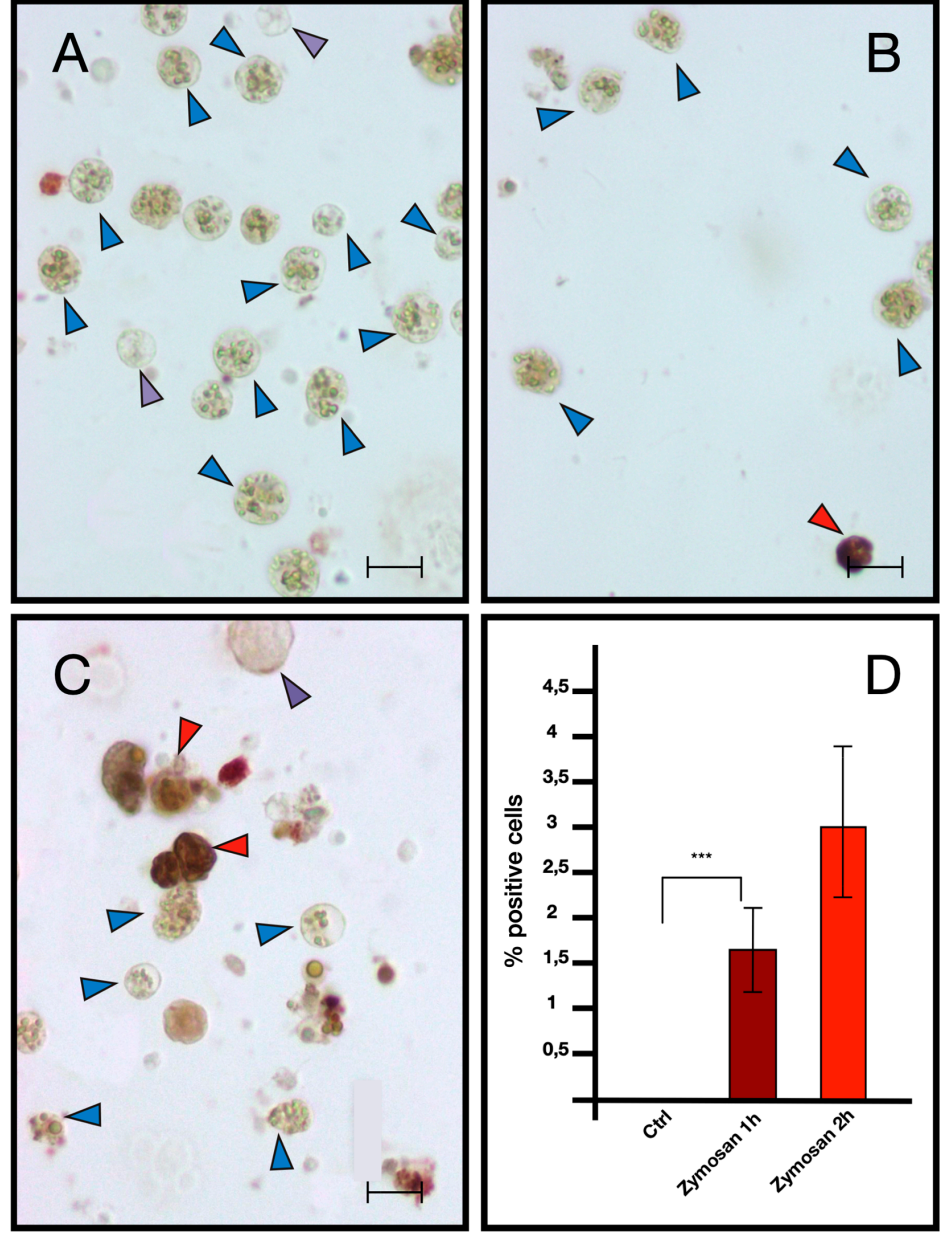
Immunocytochemistry with anti-C9 antibody on hemocytes treated with 0.1 mg/mL Zymosan for 1 and 2 h. (**A**) Control in FSW (**B**) Treated for 1 h with Zymosan 0.1 mg/mL; (**C**) treated for 2 h with Zymosan 0.1 mg/mL; (**D**) percentage of marked hemocytes. Red arrow: marked morula cells, blue arrow: unmarked morula cells, purple arrow: phagocytes. Scale bar: 10 μm. *** *p* < 0.001.

Through ISH, we showed that MCs, in addition to *bsc3*, are the only hemocytes transcribing *bsitccp* and synthesizing the protein, as demonstrated by the immunopositivity to the anti-hC9 antibody (Figure 13). In line with previous results [3,6,15,18], our data confirm that MCs, the cytotoxic circulating hemocytes of *Botryllus*, are the cells responsible for the synthesis and secretion of all the components of the complement system known so far.

In addition, we provided evidence that the transcription of *bsitccp* can be influenced by BsC3. We observed a temporal correlation between the regulation of these two genes: in the presence of nonself, an increase in the transcription of *bsc3* is observed as early as 15 min, reaching a plateau at 45 min, whereas *BsITCCP* increases its expression only at 60 min. Immunocytochemical assays confirm that the increase in BsITCCP-positive cells continues up to 2 h, while the fraction of BsC3-positive cells remains constant. In addition, the fraction of cells labeled by the antisense probes for *bsc3* and *bsitccp* collapsed after the combined treatment with zymosan and the anti-hC3 antibody as well as in the presence of compstatin, an inhibitor of C3 activation [17]. Taken together, these data depict a framework where the transcript for a protein similar to vertebrate C9 is directly modulated by *bsc3,* likely through the C3a/C5a receptor previously described in our earlier study, supporting the autocrine activity of BsC3 on MCs previously observed [15]. On this basis, it is possible to hypothesize that BsC3, the protein representing the central component of the complement system in *B. schlosseri* as well as in vertebrates, may modulate the expression of a protein that, possessing a MACPF domain, might be able to lyse target cells through the formation of pores in cell membranes. Although, up to now, there is no clear evidence of cell lysis events consequent to the recognition of nonself in *B. schlosseri*, we propose that the secretion and polymerization of BsITCCP might occur in a manner similar to that reported for PRF1 in the natural killer cells of vertebrates, i.e., through the formation of an immunological synapse [25,26]. Electron microscopy images of *B. schlosseri* immunocytes incubated with *Saccharomyces cerevisiae* showed yeast cells with perforated walls and MCs contacting the surface of yeast cells, with a confined space recalling that created by vertebrate immunocytes and in which BsITCCP could intervene in a possible complement-mediated lytic pathway.

In conclusion, we think that the idea that the lytic pathway requires the presence of a C5 orthologue is not correct as C5 appeared, in the course of vertebrate evolution, together with C4, as a duplicate of C3 following the whole genome duplications that characterize this subphylum. Thus, based on its molecular characteristics, C3 could easily perform the same role as C5 in invertebrates by inducing the expression of the pore-forming protein and potentially mediating its attachment to the membrane.

Future experiments will certainly focus on the attempt to highlight the presence of BsITCCP in the immunological synapse and to incontrovertibly demonstrate that BsITCCP can oligomerize to form a pore in cell membranes. As of today, however, we feel confident in affirming that a terminal complement complex, also known as a lytic complement pathway, exists in invertebrates because there is a protein with probable lytic activity governed by a C3 ortholog.

## 4. Materials and Methods

### 4.1. Animals

Colonies of *B. schlosseri* were collected from the Venice Lagoon, near the Marine Biological Station of the Department of Biology, University of Padova, located in Chioggia. They were transferred to glass slides and maintained in aquaria at a temperature of 18 °C, and fed with Liquifry Marine (Liquifry Co., Dorking, UK) and algae (*Tetraselmis* sp.). Colonial zooids are grouped in star-shaped systems embedded in a common tunic. Colonies include three generations of zooids: filtering adult zooids, their palleal buds, and budlets on buds. They undergo recurrent, weekly developmental changes or takeovers that define the blastogenetic cycles [27,28]. Zooids, buds, and budlets are connected by a network of vessels within the common tunic that ensure the synchronous development of the three generations [29,30]. The circulating cells include phagocytes and cytotoxic MCs, which serve as the primary immunocytes. Phagocytes, which can be amoeboid or round, make up about 20–40% of these cells. MCs, which are the predominant type of hemocyte, comprise 40–60% of the circulating cells, playing a crucial role in inflammatory responses [31].

### 4.2. Hemocyte Collection

Hemocytes were extracted using a glass micropipette after puncturing, with a fine tungsten needle, the marginal vessel in colonies previously rinsed in 0.38% Na-citrate (Sigma-Aldrich, St. Louis, MO, USA) dissolved in filtered seawater (FSW). The collected hemolymph was then centrifuged at 800× *g* for 10 min. Pelleted cells were then resuspended in FSW to reach a concentration of 5 × 10^5^ cells/mL.

### 4.3. Microinjections

Microinjections were performed on colonies of a single system using a microinjector (Leica Microsystems). Colonies were injected in marginal ampullae (the blind endings of the vasculature), with a suspension of 0.1 mg/mL zymosan (Sigma-Aldrich, St. Louis, MO, USA) in FSW, or with 0.1 mg/mL zymosan in FSW containing anti-hC3 antibody (Quidel, San Diego, CA, USA, diluted 1:200, according to the manufacturer’s instructions). Phenol red was added to the medium in order to mark the fluid and allow visualization of the injection. FSW was used for injection control experiments. RNA was then extracted 6 h after the injection.

### 4.4. Primer Design, RNA Extraction, cDNA Synthesis, Cloning and Sequencing

The sequence of a transcript showing high identity with the vertebrate transcripts for C6 or C9, referred to as BsITCCP (*Botryllus schlosseri* Invertebrate Terminal Complement Complex Protein), was identified through BLAST analysis of a *B. schlosseri* EST collection obtained in our laboratory [27] and the database of the *B. schlosseri* genome (http://botryllus.stanford.edu/botryllusgenome/; accessed on date 23 November 2023). They were elongated to full length through 5′ and 3′ RACE using the primers reported in Table S2.

Total RNA was extracted from hemocytes of *B. schlosseri* using the NucleoSpin RNA XS kit (Macherey-Nagel, Düren, Germany). The quality of the RNA was assessed based on the A_260/280_ ratio. RNA integrity was verified by visualizing rRNAs on 1.5% agarose (Sigma-Aldrich, St. Louis, MO, USA) gels stained with Midori green (Nippon Genetics, Tokyo, Japan). For the reverse transcription of the first cDNA strand, 1 µg of total RNA was incubated at 42 °C for 60 min in 20 µL of the reaction mix (1 µL of ImPromII Reverse Transcriptase (Promega, Madison, WI, USA) and 0.5 µg of either oligo(dT)-Anchor primer or random primers (Promega, Madison, WI, USA). PCR amplifications were performed in a 25 µL reaction volume that contained 100 ng of cDNA from *B. schlosseri* hemocytes, 2.5 µL of incubation buffer (PCRBIO Classic Taq, PCR Biosystems, London, UK) with 15 mM MgCl_2_, 0.25 µM of each primer, 10 mM of each deoxynucleotide triphosphate, and 2 units of Taq polymerase. The PCR protocol followed in a MyCycler (Bio-Rad, Hercules, CA, USA) thermocycler included an initial denaturation at 94 °C for 2 min, followed by 40 cycles at 94 °C for 30 s, annealing at 55–60 °C for 30 s, extension at 72 °C for 40 s, and a final elongation at 72 °C for 10 min. The resulting amplicons were run on a 1.5% agarose gel, and the bands were isolated using the ULTRAPrep Agarose Gel Extraction Mini Prep kit (AHN Biotechnologie, Nordhausen, Germany). These were then ligated into pGEM-T Easy Vector (Promega, Madison, WI, USA) and cloned into DH-5α *Escherichia coli* cells. Positive clones were identified and sequenced at Eurofins Genomics (Ebersberg, Germany) using the ABI 3730XL DNA Analyzer (Thermo Fisher Scientific, Waltham, MA, USA) as per the Sanger sequencing method.

### 4.5. Quantitative Real-Time PCT (qRT-PCR)

To estimate the total amount of mRNA for BsITCCP and BsC3, we carried out qRT-PCR with the SYBR green method (QPCRBIO sygreen mix separate rox, PCR Biosystems, London, UK). mRNA was extracted from colonies of *B. schlosseri* microinjected with 0.1 mg/mL zymosan for 15, 30, 45, and 60 min and 2 and 6 h, with or without the addition of anti-hC3 antibody. Control samples were incubated with FSW for the same times. Forward and reverse primers for the above-reported transcripts and for the elongation factor (Table S2) were synthesized by Sigma-Aldrich, St. Louis, MO, USA. The elongation factor was used as a housekeeping gene due to its stable expression. To exclude contamination by genomic DNA, all the designed primers contained parts of contiguous exons; a qualitative PCR was also carried out before qRT-PCR. Furthermore, analysis of the qRT-PCR dissociation curve gave no indications of the presence of contaminating DNA.

qRT-PCR analyses were performed using an Applied Biosystem (Applied Biosystems, Foster City, CA, USA) 7900 HT Fast Real-Time PCR System, using the following cycling parameters: 3 min at 95 °C (denaturation), 20 s at 95 °C plus 15 sec at 60 °C 45 times. Each set of samples was run three times and each plate contained cDNA from three different biological samples (*n* = 3) and negative controls. The 2^−DDCT^ method [32] was used to estimate the total amount of mRNA. The amounts of transcripts in different conditions were normalized to the elongation factor to compensate for variations in the amounts of cDNA and expressed as “fold induction”.

### 4.6. In Situ Hybridization (ISH)

Biotin-labeled antisense riboprobes for *bsitccp* and *bsc3* were obtained using the primers reported in Table S2. Hemocytes, collected as reported above, were left to adhere on SuperFrost Plus (Menzel-Gläser, Braunschweig, Germany) glass slides for 30 min. They were then incubated for 15, 30, 45, and 60 min or 2 h in FSW in the presence or absence (control) of 0.1 mg/mL zymosan and anti-hC3 antibody, washed in FSW, and fixed in 4% paraformaldehyde plus 0.1% glutaraldehyde in 0.4 M cacodylate buffer containing 1.7% NaCl and 1% sucrose, at 4 °C for 30 min, before use in either ISH or immunocytochemical protocols. Cells were then permeabilized in a solution of 0.1% Triton X (Sigma-Aldrich, St. Louis, MO, USA ) in phosphate-buffered saline (PBS: 1.37 M NaCl, 0.03 M KCl, 0.015 M KH_2_PO_4_, 0.065 M Na_2_HPO_4_, pH 7.2 from Sigma-Aldrich, St. Louis, MO, USA) for 5 min, washed in PBS, preincubated for 1 h in Hybridization Cocktail 50% formamide (Amresco, Solon, OH, USA) at 58 °C and finally hybridized overnight in the same solution containing 1 µg/mL riboprobe, at the same temperatures reported above. The reaction was blocked by two washes in SSC (0.3 M NaCl, 40 mM sodium citrate, pH 4.5), three washes in a solution of 50% formamide in SSC at 58 °C for 30 min, and two washes in PBS containing 0.1% Tween 20 (Sigma-Aldrich, St. Louis, MO, USA) (PBST) at room temperature, for 5 min. Samples were then incubated in 1% powdered milk in PBST for 1 h followed by 30 min in 5% methanol to block endogenous peroxidases and 30 min in Vectastain ABC (Vector Laboratories, Burlingame, CA, USA) in PBS. Cells were finally incubated in 0.025% 3,3′-diaminobenzidine (Sigma-Aldrich, St. Louis, MO, USA; DAB) and 0.004% H_2_O_2_ (Sigma-Aldrich, St. Louis, MO, USA in PBS for 15 min. Slides were then washed in distilled water and mounted in Eukitt (Electron Microscopy Sciences, Hatfield, PA, USA). The percentage of positive immunocytes was estimated by counting at least 200 cells per slide, in 10 optic fields at a magnification of 1000×.

### 4.7. Immunocytochemical Assays and Immunoblot Analysis

Fixed hemocytes (see above) were incubated for 30 min in 3% hydrogen peroxide in methanol to block endogenous peroxidase activity. After washing in PBS, they were treated for an additional 30 min with 3% powdered milk in PBS to prevent non-specific binding. The cells were then incubated overnight with polyclonal anti-hC3 antibody (50 μg/mL in PBS) and polyclonal anti-hC9 antibody (Quidel, San Diego, CA, USA, 10 μg/mL in PBS). Following incubation, the cells were washed and treated for 30 min with rabbit anti-goat secondary antibody conjugated with biotin. Subsequently, they were incubated for 30 min with the ABC complex (Vector Laboratories, Burlingame, CA, USA) followed by 10 min in DAB and H_2_O_2_ to reveal positive sites. The percentage of positive immunocytes was estimated by counting at least 200 cells per slide, in 10 optic fields at a magnification of 400×.

To validate the anti-hC9 antibody on colony homogenates, immunoblot analysis was performed, while the anti-C3 antibody was already validated in a previous publication [18]. Briefly, colonies (2–3 systems in size) were transferred into lysis buffer (50 mM Tris-HCl, 0.25 M sucrose, 1% SDS, 1 mg/mL pepstatin, 1 mg/mL leupeptin, 40 mg/mL PMSF, 2 mM Na-orthovanadate, 10 mM NaF, 0.1% NP-40, 5 mM EDTA, 5 mM N-ethylmaleimide), sonicated at 4 °C using a Branson 1200 sonifier (Branson, Brookfield, CT, USA) at 50% duty cycles for 1.5 min, and centrifuged at 10,000× *g*. The protein content of the supernatants was determined using the Bradford method. SDS polyacrylamide gel electrophoresis was performed according to Laemmli. Human plasma served as the source of hC9. Each well of the gel received a quantity of supernatant equivalent to 10 μg of protein. Gels were run at 10 mA/gel for approximately 3.5 h and stained with Coomassie blue. Proteins were transferred to a 0.45 µm nitrocellulose membrane (BDH) according to Towbin et al. [33], using 25 mM Tris, 160 mM glycine, 20% methanol, and 0.7 mM SDS as a transfer buffer. After blotting, membranes were washed thoroughly in Tris-buffered saline (TBS: 50 mM Tris-HCl, 150 mM NaCl, pH 7.4), incubated for 30 min in TBS containing 5% powdered milk (Sigma-Aldrich, St. Louis, MO, USA), and probed overnight with 10 μg/mL anti-hC9 polyclonal antibodies. Following extensive washing in TBS containing 0.05% Tween 20 (TTBS), membranes were incubated for 1 h with goat anti-rabbit IgG conjugated with peroxidase (Bio-Rad, Hercules, CA, USA), diluted 1/10,000 in TTBS. Immunogenic bands were revealed by incubation for 10 min in a solution of Amplite™ blue (Avantor, Radnor, PA, USA) and H_2_O_2_ as reported above. In controls, the anti-hC9 antibody was pre-absorbed overnight, at 4 °C, with an equal volume of hemocyte homogenate.

### 4.8. Electron Microscopic Analysis

Hemocytes incubated with yeast for 1h were pelleted by centrifugation; fixed in 2% glutaraldehyde in 0.2 M Na-cacodylate buffer, pH 7.2, for 2 h at 4 °C; and dehydrated in an increasing ethanol series and embedded in Epon (Fluka, Buchs, Switzerland). Ultrathin sections (60–70 nm) were stained with uranyl acetate and lead citrate. They were finally observed under a FEI TECNAI 12 transmission electron microscope (Thermo Fisher Scientific, Hillsboro, OR, USA; TEM), at 75 kV, equipped with a TIETZ high-resolution digital camera (TVIPS, Gauting, Germany).

### 4.9. BsITCCP Sequence Characterisation and Phylogenetic Analysis

The architecture domain organization of BsITCCP was investigated using SMART Version 9 [34] and Clustal Omega [35], in both cases with default settings for multiple alignments.

Alignments for the phylogenetic analysis were performed with MUSCLE software [36] with default settings, and assessed using molecular evolutionary genetics analyses (MEGA) program version 7 [37]. With this program, we evaluated different amino acid substitution models and found that the WAG + G + I was the best fit for our dataset, with the lowest Akaike Information Criterion (corrected AIC scores) = 20568.741 and maximum likelihood value (lnL) = −10196.09392. The maximum likelihood (ML) [38] method was used to build phylogenetic trees with MEGA 7. The non-distance-based phylogeny reconstruction neighbor-joining (NJ) [39] and the maximum parsimony (MP) [40] methods were also used to build phylogenetic trees. The nonparametric bootstrap test [41], with 10,000 replicates, was used to assess the robustness of the tree topologies. Sequences used for phylogenetic analysis, found in various databases, are reported in Figure S2.

### 4.10. Structure Prediction of BsITCCP

We used the AlphaFold2 software version 2.3.2 [42] to predict the tertiary structure of the BsITCCP protein. This method was run using the monomer preset in conjunction with the full collection of genetic databases distributed by the authors.

We retrieved the structure of the human C9 monomer and of the human PRF1 from SWISS-MODEL (accession 6h04.1.H) and performed a structural alignment with BsITCCP using the TM-align method available on the RCSB PDB website.

### 4.11. Statistical Analysis

The ISH and immunocytochemistry assay were performed in triplicate under the same conditions. Statistical comparisons between the fractions of labeled and unlabeled cells were conducted using the χ^2^ test. For real-time PCR analyses, which were also performed in triplicate under the same conditions, statistical comparisons were conducted using Duncan’s multiple range test (ANOVA) and Student’s *t*-distribution.

## Data Availability

The data that support the findings of this study are available from the corresponding author upon reasonable request. All the sequences are registered in GeneBank.

## Supplementary Materials

The following supporting information can be downloaded at: https://www.mdpi.com/article/10.3390/ijms252211995/s1.
