# Supplementary material for: Evidence of a Lytic Pathway in an Invertebrate Complement System: Identification of a Terminal Complement Complex Gene in a Colonial Tunicate and Its Evolutionary Implications
Source: Int J Mol Sci. 2024 Nov 8;25(22):11995. doi: 10.3390/ijms252211995 (PMC11593599; doi:10.3390/ijms252211995)
Supplement: Supplementary file 1 [file ijms-25-11995-s001.zip › Figure S1 - BsITCCP sequences.pdf]

>BsC9 mRNA

TTGTATTAAGCAGATTAGGTTAAATACTGAAGACAACGTACCAAGATCTACGGAAGCGTCAGAGCAAAATTAT
TTCCAAGGAAATGGTGGAATCGAGGATACTTCTTTTCTTCAGCGCGGTTGCATTCGTGGACTTGACGTCGCTT
GCGGCGGCCAAACGGAATTGCACGGCTACTGTCCCAGACGATTTCGGAGTATGCTTCGAAGATCTTGCTTCCG
GAACCCTGTGGCAGGAGAAGTTTATTACACTGGAGCCTGCTGACAATGGCACCATAGGGAACTGTGAAAACTA
CCGCGTCAATCAAGTGGAAAAACAGGTCTGTTCAACGCCGGTGGAAACAATTGAAGATTATCGATCTTCAAAT
TGCGGCACGTCGGGTAATTGGTATCAATGTTCGAATCGGCGATGCATACCGACGTCTTTGCAGTGCAATGGAG
ATGATGATTGCGGAGATGCGTCTGATGAGTATCCGTCTGTTAATCGACAATCCTACAATTGCACGAATCGGGG
TGAATTTATTTGTGGAGTTCCTGTAACGACCAGCGCTAGCGCCACACCCTCCAAATCTGATCGATATTTTCTG
GGGATTCCAGGATTGGAAAATTTAGCGTCAGGATTCGACATTCTAACCGGGACTCGCAAAGGTGACGTCTTAT
CTATGGGCCCGCACGGATCGTGCAGGAGAGTTCTAAAACATGGACTGGACGATTATTACTACCGATTACCAAA
CAACATCAATTCCCTGACACAGCTGTTTTCCGTCGACGAAGCTCCCGGAGAGATCGTCTCCACGGCGGCAGAA
TATTTGAAGTTGAGCGAAGATATGTTGGCACTTGATTATACTTTTGGAGGATACCTTAATAATGACGACAGTG
CGATGGGCCTGACTCAATCTGAGAAAACCAAGTCGTTTTCGTTAGCAAATATAGATACGCAAAATCGGTACGT
GGCAGTTACAACCACGAACAGGGTCAACGTTTTCAAGGTGGAATTGAGATCCCCGCGAAACTTGGAAGCCTCG
AATTCATTCCTTGGGCGACTTTACGGACTGCCTCGGGATAATTTTAGTTACGACCGCTATCTTGCCTTCTTGC
GAGATTTTGGAACGCATTATTTCTCATCGGGTGTACTTGGAGGACAATATACTTCTGTTAATCTCTATGATCG
CTGCTGGATTGATAGCGAATTGGAACAAACCCTAGCATTAACTCGCACAGAGTGGCTGGAAAAATTAGCCTTC
TGCGACCGACAAAGCGTAAACAACATACTCAATCCCACTTACGAAATTCCATCATCGTGCATCCTGGGAGCTT
CTACGACTTCTACAGGAGTGAACATCGCTGAAAATGCAATCAAAACCGATGTCACCGTCATGGGAGGTTCTGT
TTCAACAACAGCAAAGCTACGAGACACCTTGAGCAGCGCCAATTGGCAAGCATGGGCTGAAACCGTTGCAGAG
ACTCCCACTTTGTTGCTTCATGATTTTGAACTTACAAGAATCTCCAAACTTCTTGATAACCCTCTTCCTATCT
TCACGAATGAGCAACGTCTGAAAGTACAACAATTTTTGGATGAAGCCATTTCGATCTATTTGAAACCATACAA
CTCCGCGTCGTGATAGTTGCTTTCTTGAAACCGAAAATACTGACAGCGTCTATTCTGCTTACCTTACCGGAAC
GTCGGGGAAGGAATATTATTGCCGTTGCGCTTGCGATAATGCGCCACGTATTGCTGATTGCAATGGATCTCAA
TCGACAAGCGCAACTTCGTACCCCACTCTCCTCGTCAATTTACTTATTAGTGTCTCCATAATTCGAAAAATTA
ATTCCATATCTGGCTAATTAATTATCGGGTACGACTCTGCTGAGTAAAAAGCGTGGACTGTCCCCGACTGTCG
GTTACAGACTGTGCGATAGCCAATGTCCAAAGACTACTTTAGTATATATATTTAGTTATCATTCGTATTTGTA
TTGGCTATTATGACAGTTGTATGTATTGCTGTCATGCGATGAATTTAAGAGTTGGTTTGCGATACTTTTGTAC
ATACTCTCTTATTCTTATAGGCTATTACCTTCCATGTTTATAGAAAAGTAAACAATTCGCGTTCACAATCCTT
GGCAATCTATACGCAGTGTGTCTATGTTGGAAACTGATACAGCAGACAATAGCGCCAATGCAATCAAACTGCT
GTGATGTGTAGTGTGCATTAACGGTCCATTAGGCCTACCGTACTTAGTGCTTATATACTTCACAGTTGACAGT
AATCAGTATTAAAGATGGTGCCGCAATATCCAAATATTTCTGATTCTTGAACGATAAAGCAGGAAAACAGCAC
TGGTGGACAAGTCCACGGACAGAGGTGGAATATATAGCCTGGTTATTATCAGAGGTGGTCAAAGTAGTCAAAG
CTACTCAAGTAGAAGTAGAAAAAAAAAAAAAAAAAAAAAAAAAAAAAAAAAAAAAA

>BsC9 protein

MVESRILLFFSAVAFVDLTSLAAAKRNCTATVPDDFGVCFEDLASGTLWQEKFITLEPADN
GTIGNCENYRVNQVEKQVCSTPVETIEDYRSSNCGTSGNWYQCSNRRCIPTSLQCNGDDDC
GDASDEYPSVNRQSYNCTNRGEFICGVPVTTSASATPSKSDRYFLGIPGLENLASGFDILT
GTRKGDVLSMGPHGSCRRVLKHGLDDYYYRLPNNINSLTQLFSVDEAPGEIVSTAAEYLKL
SEDMLALDYTFGGYLNNDDSAMGLTQSEKTKSFSLANIDTQNRYVAVTTTNRVNVFKVELR
SPRNLEASNSFLGRLYGLPRDNFSYDRYLAFLRDFGTHYFSSGVLGGQYTSVNLYDRCWID
SELEQTLALTRTEWLEKLAFCDRQSVNNILNPTYEIPSSCILGASTTSTGVNIAENAIKTD
VTVMGGSVSTTAKLRDTLSSANWQAWAETVAETPTLLLHDFELTRISKLLDNPLPIFTNEQ
RLKVQQFLDEAISIYLKPYNSASFCNCNDSCFLETENTDSVYSAYLTGTSGKEYYCRCACD
NAPRIADCNGSQSTSATSYPTLLVNLLISVSIIRKINSISG

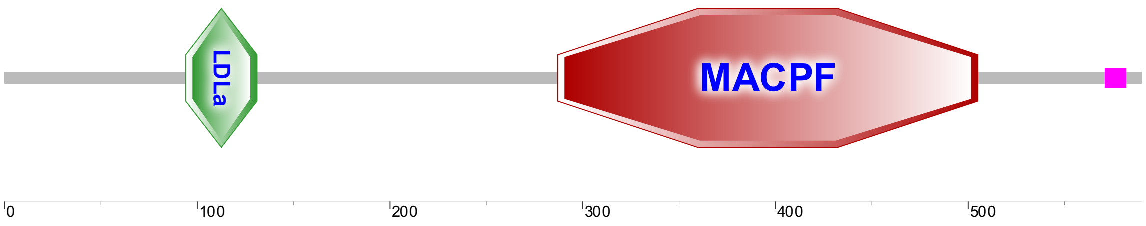
LDLa
MACPF
0
100
200
300
400
500

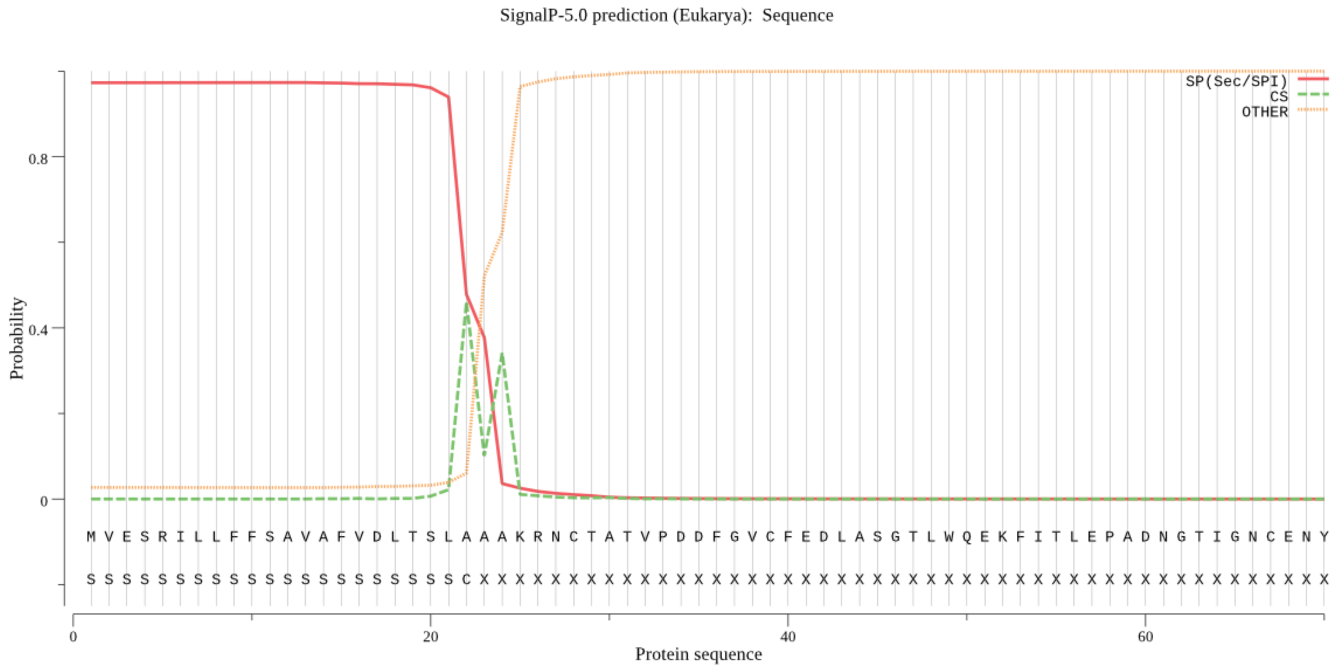
SignalP-5.0 prediction (Eukarya): Sequence
SP(Sec/SPI)
CS
OTHER
Probability
0.8
0.4
0
M V E S R I L L F F S A V A F V D L T S L A A A K R N C T A T V P D D F G V C F E D L A S G T L W Q E K F I T L E P A D N G T I G N C E N Y
S S S S S S S S S S S S S S S S S S S S S C X X X X X X X X X X X X X X X X X X X X X X X X X X X X X X X X X X X X X X X X X X X X X X X X X
0
20
40
60
Protein sequence
