# Supplementary material for: Evidence of a Lytic Pathway in an Invertebrate Complement System: Identification of a Terminal Complement Complex Gene in a Colonial Tunicate and Its Evolutionary Implications
Source: Int J Mol Sci. 2024 Nov 8;25(22):11995. doi: 10.3390/ijms252211995 (PMC11593599; doi:10.3390/ijms252211995)
Supplement: Supplementary file 1 [file ijms-25-11995-s001.zip › Figure S2 - Extended PhylogeneticTree.pdf]

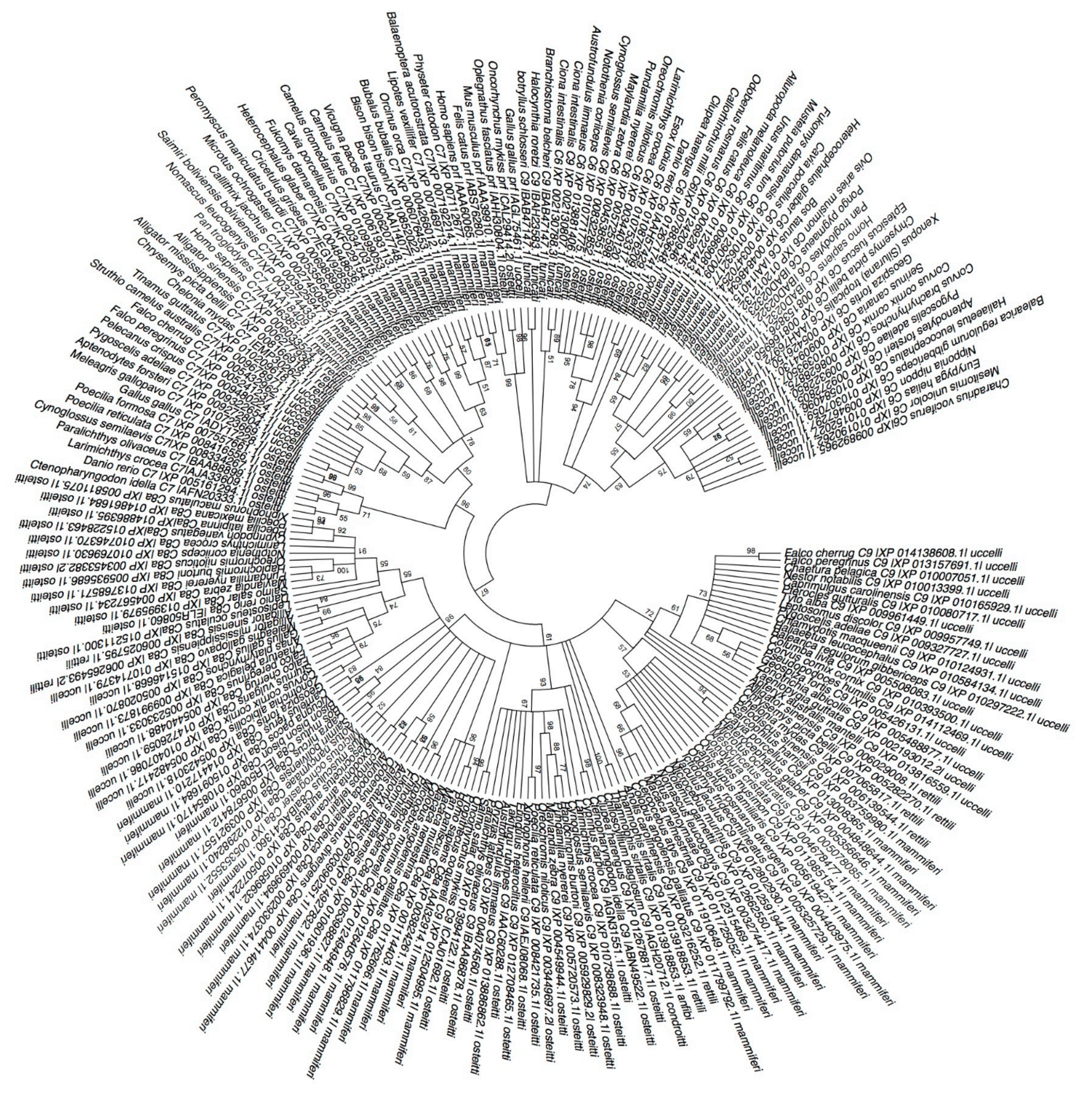

Sup 4 - Phylogenetic tree of the MACPF domain for C6, C7, C8, C9, and perforin proteins, calculated using the Maximum Likelihood method with 2000 Bootstrap replicates. Full names
