# Supplementary material for: Evidence of a Lytic Pathway in an Invertebrate Complement System: Identification of a Terminal Complement Complex Gene in a Colonial Tunicate and Its Evolutionary Implications
Source: Int J Mol Sci. 2024 Nov 8;25(22):11995. doi: 10.3390/ijms252211995 (PMC11593599; doi:10.3390/ijms252211995)
Supplement: Supplementary file 1 [file ijms-25-11995-s001.zip › Table S2 - Species list by phylogeny.pdf]

Species	C6	C7	C8	C9
Mus musculus	AAF14577.1		BAC41360.1	
Pongo pygmaeus	BAD02323.1			
Oplegnathus fasciatus	AIN76757.1			
Pan troglodytes	BAD02322.1	JAA18409.1		
Homo sapiens	BAD02321.1	AAH63851.1	AAI32914.1	
Bos taurus	AAI14015.1	AAI14077.1	AAI12636.1	
Microtus ochrogaster	XP_005356638.1	XP_005356640.1	XP_005353552.1	XP_005356646.1
Cavia porcellus	XP_003470254.1	XP_003470315.1		XP_013008365.1
Heterocephalus glaber	XP_004848634.1	XP_004848636.1		XP_004848644.1
Jaculus jaculus	XP_004652555.1		XP_012807492.1	XP_012802930.1
Fukomys damarensis	XP_010632177.1		XP_010601936.1	
Mustela putorius furo	XP_012907009.1		XP_004751679.1	
Odobenus rosmarus	XP_012420946.1		XP_004414677.1	XP_004403975.1
Felis catus	XP_006928145.1		XP_003990251.1	
Ailuropoda melanoleuca	XP_011227443.1		XP_002930374.1	
Ursus maritimus	XP_008690814.1			
Pteropus vampyrus	XP_011368981.1		XP_011368719.1	
Eptesicus fuscus	XP_008153826.1			
Ovis aries musimon	XP_012012132.1		XP_011982512.1	XP_011985154.1
Echinops telfairi	XP_004716995.1			
Trichechus manatus latirostris	XP_004388494.1			
Ochotona princeps	XP_004583839.1		XP_012783446.1	
Cercocebus atys		XP_011910670.1	XP_011937888.1	XP_011910649.1
Macaca nemestrina		XP_011749193.1	XP_011762666.1	XP_011725052.1
Colobus angolensis palliatus		XP_011799808.1	XP_011796829.1	XP_011799792.1
Mandrillus leucophaeus		XP_011821752.1	XP_011829235.1	
Rhinopithecus roxellana		XP_010380072.1	XP_010364034.1	
Saimiri boliviensis boliviensis		XP_003925947.1	XP_003921557.1	
Callithrix jacchus		XP_002745085.2		
Nomascus leucogenys		XP_003274433.1	XP_003265183.1	XP_003274417.1
Cricetulus griseus		EGV92855.1		
Peromyscus maniculatus bairdii		XP_006988992.1		
Fukomys damarensis		KFO29154.1		
Capra hircus		XP_005694811.1	XP_005678412.1	
Bison bison bison		XP_010852022.1	XP_010854170.1	
Bubalus bubalis		XP_006076453.1		
Orcinus orca		XP_004266012.1	XP_004273844.1	
Lipotes vexillifer		XP_007469713.1		
Balaenoptera acutorostrata scammoni		XP_007192214.1		
Physeter catodon		XP_007112677.1		
Camelus dromedarius		XP_010999053.1	XP_010982881.1	
Camelus bactrianus		XP_010955628.1	XP_010956668.1	
Vicugna pacos		XP_006207748.1	XP_015091684.1	
Camelus ferus		XP_006193113.1	XP_014417210.1	
Macaca fascicularis			XP_005543304.1	
Microcebus murinus			XP_012640576.1	XP_012591944.1
Bos mutus			ELR50860.1	

Macaca mulatta			XP_001114403.1	
Acinonyx jubatus			XP_014927832.1	
Equus asinus			XP_014700167.1	
Tupaia chinensis			XP_006146161.1	
Myotis brandtii			XP_014398054.1	
Pan paniscus			XP_003824261.1	
Canis lupus familiaris			XP_003639070.1	XP_005619427.1
Chinchilla lanigera			XP_005398827.1	
Ictidomys tridecemlineatus			XP_013214378.1	XP_005325729.1
Mesocricetus auratus			XP_005072241.1	XP_005078085.1
Dipodomys ordii			XP_012877719.1	
Sorex araneus			XP_004607361.1	
Otolemur garnettii			XP_003793169.1	XP_003793037.2
Condylura cristata			XP_004679102.1	XP_004678477.1
Propithecus coquereli			XP_012494948.1	XP_012504995.1
Taeniopygia guttata			XP_002194495.2	XP_002193012.2
Sarcophilus harrisii			XP_012403037.1	XP_003759980.1
Aotus nancymaae			XP_012298240.1	XP_012315469.1
Loxodonta africana			XP_010589632.1	
Geospiza fortis	XP_005426150.1		XP_005423018.1	XP_005426131.1
Zonotrichia albicollis	XP_005488884.1			XP_005488877.1
Taeniopygia guttata	XP_002193277.2			
Corvus cornix cornix	XP_010393507.1		XP_010407086.1	XP_010393500.1
Serinus canaria	XP_009094130.1			
Manacus vitellinus	XP_008919868.1			
Corvus brachyrhynchos	XP_008628875.1			
Falco cherrug	XP_005432984.1	XP_005432962.1		XP_014138608.1
Haliaeetus leucocephalus	XP_010584096.1	XP_010584097.1		XP_010584134.1
Phaethon lepturus	XP_010282305.1			
Egretta garzetta	XP_009645626.1	XP_009645696.1		
Nipponia nippon	XP_009467297.1	XP_009467296.1		
Pelecanus crispus	XP_009491752.1	XP_009480224.1		
Balearica regulorum gibbericeps	XP_010307059.1			XP_010297222.1
Chlamydotis macqueenii	XP_010128161.1			XP_010124931.1
Mesitornis unicolor	XP_010190262.1		XP_010180633.1	
Eurypyga helias	XP_010152027.1			
Cariama cristata	XP_009694100.1			
Pygoscelis adeliae	XP_009322850.1	XP_009322654.1	XP_009318073.1	XP_009327727.1
Aptenodytes forsteri	XP_009272805.1	XP_009272804.1		
Merops nubicus	XP_008947283.1	XP_008941053.1		
Melopsittacus undulatus	XP_005152162.1	XP_005152034.1		
Nestor notabilis	XP_010013798.1			XP_010013399.1
Struthio camelus	XP_009678974.1	XP_009678971.1		
Charadrius vociferus	XP_009892965.1			
Cuculus canorus	XP_009559654.1	XP_009559655.1		
Tinamus guttatus	XP_010209619.1	XP_010209618.1		
Pterocles gutturalis	XP_010075199.1		XP_010077278.1	XP_010080717.1
Caprimulgus carolinensis	XP_010176089.1			XP_010165929.1

Apaloderma vittatum	XP_009872948.1			
Apteryx australis mantelli	XP_013816476.1			XP_013816559.1
Gallus gallus		ADY17228.1	XP_015146668.1	
Phalacrocorax carbo		XP_009509566.1		
Falco peregrinus		XP_005241237.1	XP_005233033.1	XP_013157691.1
Leptosomus discolor		XP_009953137.1		XP_009957749.1
Meleagris gallopavo		XP_010723669.1	XP_010714379.1	
Chaetura pelagica		XP_010007012.1	XP_009991873.1	XP_010007051.1
Picoides pubescens		XP_009898166.1		
Calidris pugnax			XP_014794629.1	
Sturnus vulgaris			XP_014726059.1	
Columba livia			XP_005498948.1	XP_005508083.1
Anas platyrhynchos			XP_005020870.1	
Aquila chrysaetos canadensis			XP_011570443.1	
Pseudopodoces humilis				XP_014112469.1
Tyto alba				XP_009961449.1
