# Supplementary material for: Evidence of a Lytic Pathway in an Invertebrate Complement System: Identification of a Terminal Complement Complex Gene in a Colonial Tunicate and Its Evolutionary Implications
Source: Int J Mol Sci. 2024 Nov 8;25(22):11995. doi: 10.3390/ijms252211995 (PMC11593599; doi:10.3390/ijms252211995)
Supplement: Supplementary file 1 [file ijms-25-11995-s001.zip › TableS1 - Primer table.pdf]

Gene name	Primer name	Sequence (5’->3’)
BsITCCP	ITCCPF2	ATGATGATTGCGGAGATGCGTCT
	ITCCPR2	GAATGTCGAATCCTGACGCT
	ITCCPF1	AACCATACAACTCCGCGTC
	ITCCPR1	CGTTCCGGTAAGGTAAGCAG
BsC3	BsC3-F	ACAAACAGGACCCGAACAAG
	BsC3-R	TGAGCGCCACATACTGTCTT
BsEF	BsEF-F	GCCGCCATACTCTGAAGC
	BsEF-R	GTCCAACTGGCACTGTTCC
